# Landscape of germline cancer predisposition mutations testing and management in pediatrics: Implications for research and clinical care

**DOI:** 10.3389/fped.2022.1011873

**Published:** 2022-09-26

**Authors:** Shilpa A. Shahani, Erin L. Marcotte

**Affiliations:** ^1^Department of Pediatrics, City of Hope Comprehensive Cancer Center, Duarte, CA, United States; ^2^Division of Epidemiology and Clinical Research, Department of Pediatrics, University of Minnesota, Minneapolis, MN, United States; ^3^Masonic Cancer Center, University of Minnesota, Minneapolis, MN, United States

**Keywords:** germline predisposition, childhood cancer, genetic testing, cancer predisposition, cancer syndrome

## Abstract

As germline genetic testing capacities have improved over the last two decades, increasingly more people are newly diagnosed with germline cancer susceptibility mutations. In the wake of this growth, there remain limitations in both testing strategies and translation of these results into morbidity- and mortality-reducing practices, with pediatric populations remaining especially vulnerable. To face the challenges evoked by an expanding diversity of germline cancer mutations, we can draw upon a model cancer-associated genetic condition for which we have developed a breadth of expertise in managing, Trisomy 21. We can additionally apply advances in other disciplines, such as oncofertility and pharmacogenomics, to enhance care delivery. Herein, we describe the history of germline mutation testing, epidemiology of known germline cancer mutations and their associations with childhood cancer, testing limitations, and future directions for research and clinical care.

## Introduction

Approximately 10,470 children younger than 15 years old will be diagnosed with cancer in the United States in 2022 (1) and it is estimated that up to 397,000 cases of childhood cancer occur in this demographic worldwide annually (2). Pediatric cancers are heterogeneous and broadly classified as hematologic malignancies, central nervous system (CNS) tumors, and non-CNS solid tumors. Within these classifications, there are numerous subtypes characterized by distinct clinical and prognostic features. While the etiologies of most childhood cancers are poorly understood, there is a growing appreciation for the role of germline genetic susceptibility, which may underlie a non-trivial proportion of cases. As characterization of germline genetic susceptibility among childhood cancer patients has improved, there is increased recognition of the unique challenges and opportunities of genetic testing, therapeutic modifications, and cancer surveillance in the pediatric population.

### Germline cancer mutations

Increasingly more genetic changes linked to cancer predisposition are being recognized due to a rapidly expanding genetic field. Cancer risk can now be attributed to whole categories of germline genetic changes including amino acid sequence mutations, translocations, trisomies, and imprinting, among others (3, 4). Types of mutations include frameshift, splice site, missense, nonsense, and silent, each of which confers varying risk of developing disease based on both the function of the affected gene and location of the genetic change along the allele. Trisomies that can survive gestation include Trisomy 13 (Patau Syndrome), 18 (Edwards Syndrome), and 21 (Down Syndrome), the last of which may also be secondary to a Robertsonian translocation (5). Additionally, certain genetic changes have differing phenotypes depending on if the mutated gene derives from the maternal vs. the paternal side, which is a function of epigenetic imprinting; a classic example is Prader-Willi Syndrome when the deletion of Chromosome 15q11 is inherited from the father and Angelman Syndrome when that same deletion is inherited from the mother (6).

### Proof of concept: Trisomy 21

Trisomy 21 (Down Syndrome) is held up as a model genetic condition for which the medical community broadly, and the field of oncology more specifically, has developed a wealth of experience in optimizing care delivery and cancer outcomes. Since 1959 (7), when Trisomy 21 was first identified, evidenced-based efforts to improve outcomes have led to decreased morbidity and mortality from enhanced supportive care strategies during standard cancer therapy and enhanced survival outcomes due to therapy modification (8, 9). While there is still much work to be done in optimizing the management of those with Trisomy 21 (10), we propose that similar attention to other genetic cancer predisposition conditions should also be undertaken and will similarly improve outcomes across a broad range of high-risk populations.

## Efforts to establish epidemiology

### Germline mutation identification

Germline genetic testing strategies have improved from diagnoses historically based solely on clinical presentation and family pedigree studies to chromosome analyses using karyotypes, whole genome and/or exome sequencing, RNA sequencing, imprinting evaluations, and heterozygosity analyses. Critical to these advancements have been the determination of a reference or normal value for each amino acid sequence in the genome; however, the reference is limited by a narrow range of ancestral diversity from the initial small pool of participants who were involved in generating the normal ranges (11). Amino acid sequences that may be physiologically normal can be classified as a germline mutation, most commonly as a Variant of Unknown Significance (VUS), because they may not be seen within the reference database. Even more worrisome are the significant portions of genomic reads that are discarded because they do not align with the reference genome, leading to potentially unrecognized pathogenic mutations. This will gradually become less likely with improved ancestral diversity in references populations (11).

Efforts to improve the diversity of the reference database and diseased populations alike are hampered by both historically decreased funding prioritization and distrust of the medical and scientific establishments among marginalized populations (12). The basis of this mistrust lies in a string of historical events and practices, including unanesthetized gynecological procedures on slave populations (13), the Tuskegee experiment (14), involuntary sterilization (15, 16), and ethno-eugenic practices (16) as well as ongoing pervasive systemic and structural racism that also manifests in the medical field with increased maternal mortality related to childbirth (17, 18), decreased recognition of pain (19), and decreased survival post cancer diagnoses across a range of malignancies (20). Efforts to remediate the medical community include recently employed diversity awareness and inclusion initiatives with the aim of reducing barriers (21) and improving medical relationships with minority populations. Within the field of genetics, specifically, efforts to improve the racial composition of the reference database include the 10,000 genome project and the National Heart, Lung, and Blood Institute (NHLBI) Trans-Omics for Precision Medicine (TOPMed), which has dramatically increased the size and diversity of this reference database (22). While data are currently lagging, there are active efforts at every level to address lack of diversity across reference and study populations, with the aim to ensure study findings are more generalizable across populations of varying ancestries.

### Limitations to identifying germline cancer predisposition prevalence across pediatric malignancies

The prevalence and burden of germline cancer mutations in pediatrics is poorly understood. Childhood cancer etiology has historically been considered primarily idiopathic, with small percentages attributed to specific causes such as environmental exposure (e.g., ionizing radiation), viral triggers (e.g., Epstein-Barr Virus), or underlying genetic predisposition (23). While it has long been recognized that certain genetic syndromes are associated with increased risk of childhood cancer, efforts to identify the prevalence of germline cancer susceptibility among childhood cancer patients are ongoing. Table 1 shows syndromes recognized to be associated with pediatric malignancies; of note, we focus here on monogenic disorders and syndromes associated with increased childhood cancer susceptibility which do not have interventions that prevent cancer development. Therefore, bone marrow failure syndromes that are cured with transplant without subsequently elevated cancer risk are beyond the scope of this article.

**Table 1:** Recognized syndromes associated with pediatrics tumors.

**Syndromes associated with pediatrics cancers**	**Tumors involved**	**Gene**
*BAP1* Syndrome	Spitz tumors, uveal melanoma, mesothelioma, clear cell renal cell carcinoma	*BAP1*
Beckwith-Wiedemann Syndrome	Wilms tumor, Hepatoblastoma	Methylation defects (50%), uniparental disomy (20%), *CDKN1C* mutation, other chromosomal abnormalities
CMMRD	Brain tumors, leukemia, gastrointestinal cancers	*MLH1, MSH2, MSH6, PMS2*
*DICER1* Syndrome	Pleuropulmonary blastoma, pituitary blastoma, thyroid cancer, ovarian tumors, cystic nephromas	*DICER1*
Denys-Drash Syndrome	Wilms Tumor	*WT1*
Down Syndrome	AML, ALL, Transient Myeloproliferative Disorder	Trisomy 21
Fanconi Anemia*	AML; squamous cell carcinomas of the head and neck, esophagus, and vulva; cervical cancer; liver tumors	*FANCA, FANCC, FANCG, FANCD2, FANCE*
Gorlin Syndrome	Medulloblastoma	*PTCH1, SUFU, GLI3*
Juvenile Polyposis Syndrome	Gastrointestinal cancers	*BMPR1A, SMAD4*
Li-Fraumeni	Adrenocorticoid carcinoma, choroid plexus carcinoma, leukemia, osteosarcoma, brain tumors	*TP53*
MEN2 syndrome	Pheochromocytoma, medullary thyroid cancer, parathyroid/pituitary tumors, carcinoids, well-differentiated tumors of gastroenteropancreatic origins	*RET*
Neurofibromatosis type 1	Leukemia, brain tumors	*NF1*
Neurofibromatosis type 2	Astrocytomas, schwannomas, meningiomas	*NF2*
Hereditary retinoblastoma	Retinoblastoma, osteosarcoma, melanoma, soft tissue sarcomas, midline PNETs	*RB1*
Tuberous sclerosis	Renal cell carcinomas, astrocytomas	*TSC1, TSC2*
Von-Hippel Lindau	Renal cell carcinoma, CNS hemangiomas	*VHL*
WAGR syndrome	Nephroblastoma	11p deletion

ALL, acute lymphoblastic leukemia; AML, acute myeloid leukemia; CMMRD, constitutional mismatch repair deficiency; MEN2, multiple endocrine neoplasia type 2; PNET, primitive neuro-ectodermal tumor; WAGR, Wilms tumor, Aniridia, Geniturinary anomalies, Range of developmental delays.

*Over 20 genes contribute to the Fanconi Anemia phenotype (24); only the genes that contribute >2% of the cases are included in this chart.

The Pediatric Cancer Genome Project (PCGP) began characterizing the prevalence of germline cancer mutations among children diagnosed with cancer who were enrolled across its clinical trials, resulting in a landmark paper published in 2015, which identified germline cancer mutations in 8.5% of cases using a cancer predisposition gene panel containing 60 genes (25). Improvements in genetics testing, including larger panels of genes tested, enhanced sequencing analyses and more robust reference databases, have more recently led to germline cancer mutations being identified among childhood cancer cases at much higher rates than previously recognized. A 2021 St. Jude study evaluated 156 cancer predisposition genes using whole genome, whole exome, and RNA sequencing, and identified pathogenic or likely pathogenic mutations in 18% (55/300) of children diagnosed with cancer, ranging from 10% among those diagnosed with a hematologic malignancy to 50% among those diagnosed with subsets of solid tumors (26). Table 2 shows select recent publications reviewing the frequency of germline cancer mutations and the corresponding number of cancer predisposition genes on their testing panels across common pediatric malignancies. As our testing platforms continue to evolve, we may discover that current estimates are lower than the true germline cancer predisposition prevalence (34).

**Table 2:** Prevalence of germline cancer mutations detected in common pediatric malignancies.

**Pediatric cancer**	**Prevalence of germline mutations detected**	**Number of genes on panel**	**References**
Leukemia	4.4% (26/588)	60	(25)
AML	19.4% (14/72)	216	(27)
Medulloblastoma	11% (76/673)	32	(28)
Hepatoblastoma	33.3% (10/33)	222	(29)
Osteosarcoma	28% (281/1,004)	238	(30)
Rhabdomyosarcoma	7.3% (45/615) 13.2% (16/121)−15.8% (44/273)	63 130	(31, 32)
Pan-solid tumors	18% (138/751)	88	(33)
Pan malignancy	18% (55/300)	156	(26)

AML, acute myeloid leukemia.

A further barrier to recognizing true cancer predisposition prevalence among those with childhood cancer lie in the study-specific selection of subjects. The majority of current analyses rely on samples collected for exploratory studies among patients included on clinical trials, most specifically those from Children's Oncology Group (COG) or from the National Cancer Institute (NCI) (30). This, by default, excludes ineligible patients, those who did not present to an institution that enrolls onto COG or NCI trials, and those who did not consent to a clinical trial. Clinical trial eligibility criteria may exclude those with known germline cancer syndromes, which will reduce the pool of germline cancer mutations in the patient samples and suggest the prevalence of germline cancer mutations in the population of those who develop childhood cancer is lower than the true prevalence. Clinical trials also consistently underrepresent racial minorities, specifically Black and indigenous populations (35), which poses a significant limitation and make the findings less generalizable. Additionally, those who reject blood transfusions are historically less likely to enroll on clinical trials, leading to patients who are Jehovah's Witnesses being underrepresented in the sample testing pool (36). While Jehovah's Witness is not a racial or ethnic group, they have a higher proportion of minority representation relative to the overall United States population (37). Racial minorities are doubly affected due to concurrent underrepresentation in both study populations and the reference database, leading to decreased sensitivity and specificity of germline testing, respectively. Given the reduced representation of minorities in the reference database, germline testing reports of non-European ancestry populations are more likely to contain VUS, which, when reported back to a participant or their family, may cause unnecessary emotional distress (38). Therefore, germline mutation testing in this population is both less accurate and carries a higher likelihood of causing harm. Determination of germline cancer predisposition prevalence based on these clinical trials becomes similarly limited.

### Features associated with increased risk of germline cancer predisposition among pediatric cancer patients

Recognized factors that increase the likelihood of germline cancer predisposition diagnoses across a pediatric oncology cohort include: presence of features on physical exam that are consistent with a known cancer-associated genetic syndrome, such as with Trisomy 21 or Beckwith-Wiedemann Syndrome; family history of cancer consistent with recognized inheritance patterns; individual history of multiple cancer diagnoses, such as osteosarcoma after a prior retinoblastoma (39); multifocal disease, such as with bilateral retinoblastoma or multifocal nephroblastoma; and treatment toxicities consistent with an underlying diagnosis, such as prolonged cytopenias with Fanconi anemia (40). Table 3 reviews select malignancies which are associated with a relatively high likelihood of underlying germline cancer predisposition. It is also important to recognize that *de novo* mutations may underlie germline cancer susceptibility in a proportion of childhood cancer cases (49) and that these cases will likely present without family histories indicative of germline cancer predisposition.

**Table 3:** Pediatric cancers with a high frequency of associated germline mutations.

**Pediatric cancer**	**Associated syndrome and gene**	**Frequency of associated germline mutations**	**References**
Adrenocorticoid carcinoma	Li-Fraumeni (*TP53*)	34/68 (50%)	(41)
Choroid plexus carcinoma	Li-Fraumeni (*TP53*)	18/36 (50%)	(42)
Malignant peripheral nerve sheath tumor	Neurofibromatosis Type 1 (*NF1*)	6/7 (85.7%)	(33)
Pheochromocytomas and paraganagliomas	Von-Hippel Lindau (*VHL*), Neurofibromatosis Type 1 (*NF1*), MEN2 syndrome (*RET*), *SDHA/B/C/D/F2, TMEM127, MAX*	66/271 (24%) to 15/36 (42%), nonsyndromic, without family history 19/24 (79%) to 10/11 (90.9%), nonsyndromic, with family history	(43, 44)
Pituitary blastoma	*DICER1* syndrome (*DICER1*)	8/10 (80%)	(45)
Pleuropulmonary blastoma	*DICER1* syndrome (*DICER1*)	11/14 (78.6%)	(46)
Retinoblastoma	Hereditary retinoblastoma (*RB1*)	47/51 (92%), with family history 115/125 (92%), bilateral 25/257 (10%), unilateral, without family history	(47)
Rhabdoid tumors	Rhabdoid tumor predisposition syndrome (*SMARCB1*)	23/65 (35%), atypical teratoma/rhabdoid tumor 3/12 (25%), renal malignant rhabdoid tumor	(48)

MEN2, multiple endocrine neoplasia type 2.

Additionally, the American Association for Cancer Research (AACR) Childhood Cancer Predisposition Workshop recommends germline testing for close to 50 specific pediatric cancer diagnoses, regardless of any other risk factor (50). Specific histological subtypes within cancers may also be suggestive of an underlying cancer predisposition, such as increased likelihood of germline *TP53* mutations among those with hypodiploid acute lymphoblastic leukemia (ALL) (51), germline *APC* mutations among those with *WNT* subtype medulloblastoma (28, 52), and germline *BRCA* mutations among those with triple negative breast cancer (53). Further research expanding the known associations between germline mutations and particular somatic subtypes can improve algorithms for germline testing in clinical settings.

With the advent of precision medicine, somatic genomics offer additional insight into the potential presence of an underlying germline cancer predisposition. Somatic tumor testing strategies vary, with karyotypes, targeted cytogenetic panels, fluorescent *in situ* hybridization (FISH), immunohistochemistry (IHC), tumor sequencing, and tumor mutational burden being the most common modalities utilized clinically. The sensitivity of each of these tests at detecting genetic changes vary, with large chromosomal anomalies such as a trisomy effectively identified by karyotype while smaller mutations may require IHC or tumor sequencing for detection, and may also depends upon the specific test, with tumor sequencing more effective than IHC at detecting small sequence variations (54). Table 4 reviews the concordance between somatic mutation identification and cancer predisposition diagnosis across a select group of malignancies and also highlights the need for further research in both adult- and pediatric-onset cancers. While it is important to recognize that the presence of a somatic mutation can suggest a germline mutation, it is also critical to appreciate that the lack of a somatic mutation does not rule out the presence of a germline mutation. Potential reasons for this discordance include biological, as may occur with reversion mutations in which a second mutation within the tumor tissue converts a pathogenic mutation to wildtype, or technical, as may occur with targeted somatic panels that are no sufficiently inclusive (26) or a return of results strategy that remove germline mutations references from somatic reports (60). Conversely, when somatic mutations are identified that are not detected in the germline, if the clinical presentation is highly suggestive of a germline mutation (e.g., positive family history or multiple personal cancers), the possibility of mosaicism must also be considered.

**Table 4:** Somatic-germline mutation concordance.

**Mutation**	**Tumor type**	**Somatic concordance with germline**	**References**
*GATA2*	MDS	7/15 (46.7%), tumor sequencing	(55)
*RUNX1*	MDS	1/3 (33.3%), tumor sequencing	(55)
*BRCA*	Ovarian carcinoma	319/1,124 (28.4%), tumor sequencing	(56)
*BRCA1*	Epitheliod ovarian carcinoma	34/36 (94.4%), tumor sequencing	(57)
*BRCA2*	Epitheliod ovarian carcinoma	10/11 (93.5%), tumor sequencing	(57)
*BRCA*	Neoplastic ascites, ovarian cancer	11/11 (100%), tumor sequencing	(58)
*MLH1*	Endometrial cancer	36/245 (14.7%), IHC	(59)
*MSH2*	Endometrial cancer	22/71 (31%), IHC	(59)
*MSH6*	Endometrial cancer	27/65 (41.5%), IHC	(59)
*PMS2*	Endometrial cancer	4/37 (10.8%), IHC	(59)

AML, acute myeloid leukemia; CMMRD, IHC, immunohistochemistry; MDS, myelodysplastic syndrome.

### Risk of germline cancer predisposition across the pediatric population

The aforementioned findings are limited to those who have already been diagnosed with cancer and cannot be extrapolated to understand the population prevalence of germline cancer mutations among all children and young adults. Assuming that the overall population frequency of germline cancer mutations in pediatrics is similar to adult data, which indicate that the most common mutations are within Lynch syndrome genes and *BRCA1/2*, affecting 1 in 279 and 1 in 400 Americans, respectively (61, 62), underrepresents the demographic as all who developed cancer and did not survive adulthood are removed from the denominator. There are no pediatric population-wide germline cancer mutation testing efforts, yet such a cross-sectional study would allow the broader pediatric oncology, precision medicine, public health, and genetics communities to better understand the true prevalence and burden of germline cancer predisposition among pediatric populations. Parallel efforts that follow these patients over their lifetime, such as the Childhood Cancer Predisposition study (NCT04511806) and the InAdvance: Surveillance, Prevention, and Interception in a Population at Risk for Cancer study (NCT05463796) would have the potential to elucidate the natural history of specific germline cancer predisposition mutations and offer insight into potential strategies that can help mitigate morbidity and mortality over the life of a previvor (defined as an individual with a genetic predisposition to cancer who has not yet developed a malignancy).

## Cancer screening and prevention

The medical community has generated significant evidence for screening measures among those with genetic syndromes associated with childhood onset of cancer, which may reduce the likelihood of cancer mortality through diagnosis of cancer at earlier stages. Several institutions have developed or are developing pediatric-specific screening guidelines for those with cancer predisposition syndromes, including AACR Pediatric Cancer Working Group, St. Jude, and COG (50, 63–72). As epidemiological studies continue to interrogate screening and intervention strategies, the guidelines will continue to evolve. However, the number of mutations for which guidelines exist for pediatric patients remains small. This is, in part, due to the relatively low incidence of pediatric cancers, low testing rates for cancer predisposition among pediatric cancer patients, low testing rates of pediatric first-degree family members of those with cancer predisposition, lack of population-wide testing across the pediatric population, and few studies assessing the impact of screening or preventative interventions in this cohort. All studies in this area are challenged by low patient numbers and require large aggregate data to provide appropriate statistical power to evaluate the potential utility of an intervention.

Furthermore, while exposure to chemotherapy and radiotherapy are independently known to increase risk of subsequent malignancies, such that guidelines exist for cancer screening recommendations for childhood cancer survivors (73, 74), the crucial intersection between genetic cancer predisposition and exposure to these therapies is not well-characterized in either pediatric or adult populations. There is clear evidence that the risk of subsequent malignancy is exacerbated among cancer survivors with germline cancer predisposition mutations (75, 76), including among survivors of childhood retinoblastoma, where those who have a germline *RB1* mutation have double the risk of secondary malignancy compared to those who do not (70). Additionally, among those who develop a subsequent malignancy, it can be challenging to determine causality, with options including a new primary malignancy, a malignancy secondary to the treatment of the first malignancy, or a combination of both. Causal assessment has implications for rationally designing interventional strategies. For example, survivors of childhood cancers with *NF1* are more likely to develop a subsequent malignancy compared with non-*NF1* childhood cancer survivors, and among survivors with *NF1*, those who were treated with radiation, but not alkylating agents, have an increased risk of developing a secondary neoplasm (75, 76). This would suggest treatment regimens that avoided radiation, if feasible, would improve long-term outcomes for childhood cancer among *NF1* patients. Data to more fully describe this space are overall lacking and are currently insufficient to provide recommendations for the management of cancer survivors with germline cancer predisposition. Survivorship groups are actively studying these questions among survivors of childhood malignancies and similar studies in adults would be very beneficial given the significantly higher patient numbers as well as the increasing numbers of adult survivors of childhood cancer. Current real-world evidence is hampered by a lack of data on germline cancer mutation status in large, cross-sectional cancer or transplant registries.

## Cancer therapy modification

The real-time identification of a germline cancer predisposition mutation for a patient with newly diagnosed cancer may have immediate impact, such as in altering the oncologic risk classification, as when an inherited bone marrow failure syndrome is identified in a newly diagnosed patient with AML; altering therapy, such as avoidance of radiation therapy as much as possible for those with Li-Fraumeni syndrome (64); or altering surveillance, such as follow-up for subsequent malignancies in patients with hereditary retinoblastoma (70) or prolonged surveillance for rhabdoid patients with germline *SMARCB1* mutations (28). Created to facilitate germline cancer predisposition testing, the McGill Interactive Pediatric OncoGenetic Guidelines (MIPOGG) phone app utilizes a combination of clinical and histological features to guide testing decisions, and has been validated by several studies (77, 78), including a nationwide study in Denmark that identified a pathogenic variant in germline cancer gene in 47.5% (94/198) of newly diagnosed pediatric cancer patients (79). While it has not systemically been studied in pediatric oncology, real-time identification of germline mutations using rapid whole genome and exome testing has untapped potential, as suggested by Rady Children's Institute for Genomic Medicine's multistate consortium, which conducts these tests on critically ill infants and, in testing over 100 families, has identified 40% of patients with a genomic diagnosis and 80% of those were able to receive diagnosis-informed care that improved outcomes (80).

The value of germline cancer predisposition diagnosis among pediatric cancer patients is highlighted in the Trisomy 21 model. By consistently diagnosing and studying cancer patients with Trisomy 21, the pediatric oncology community has developed depth of expertise in germline-informed oncology care. Children with Trisomy 21 are at an increased risk of hematologic malignancies and, through dedicatedly studying this cohort, survival outcomes have improved such that they have similar rates of survival for childhood ALL (8) and higher rates of survival for acute myeloblastic leukemia (AML) relative to non-Trisomy 21 patients (9). We also understand the natural history of transient myeloproliferative disorder, which may occur among Trisomy 21 infants (81), and have developed guidelines for which patients need chemotherapy, how to deliver chemotherapy effectively to those who need it (82, 83), and how to follow them for future development of AML. Additionally, we have recognized an increased toxicity to standard chemotherapy regimens (84, 85), leading to reduced dosing of methotrexate (86), increased utilization of leucovorin (87), prophylactic antiepileptics during immunotherapy (88), reduced anthracycline administration (89), increased prophylactic antimicrobial usage and suggested hospitalizations during periods of significant immunocompromised states (90). Ultimately, we have dedicated protocols, in the case of AML (89), or arms of protocols, in the case of ALL (NCT03914625, NCT04546399), for children with Trisomy 21. Given the depth of expertise we have with Trisomy 21, we are also able to offer effective multidisciplinary care that addresses comorbidities or high-risk adverse events, such as concurrent management of congenital heart disease or screening for development of cataracts (91), respectively, and tailored post-cancer surveillance (91, 92).

We have demonstrated the capacity to overcome systemic barriers and enhance cancer outcomes for children with Trisomy 21. Thus, the medical and scientific communities can and should develop a similar expertise in tailoring oncology therapy across all cancers diagnosed among those with germline cancer predisposition. Indeed, we are already developing the tools to do so in the parallel, though far more advanced, movement occurring in the somatic space, for which exists a whole therapeutic landscape that target specific genetic mutations. In this era of precision medicine, the somatic genetic profile of almost all cancers has implications on their treatment and outcomes. A resultant paradigm shift allows us to view cancers grouped by common somatic biology, enabling targeting across malignancies and paving the way for biomarker-driven clinical trials (93).

Germline profiling allows another perspective shift, in which cancers may be grouped by a common germline mutation. Exciting work in this space is occurring with the advent of poly adenosine diphosphate-ribose polymerase (PARP) inhibitors in the management of breast (NCT05141708, NCT03150576), prostate (NCT04030559, NCT03442556), and pancreatic cancer (NCT04858334, NCT04548752) for those with germline *BRCA* mutations. Table 5 shows interventional trials for cancer treatment among patients with germline cancer mutations. As a surrogate, elevated tumor microsatellite instability (MSI) is often associated with Lynch Syndrome and numerous studies are evaluating the efficacy of immune checkpoint inhibitors across multiple tumor types that express a high MSI. This arena remains in its infancy as these evaluations are just beginning for cancers associated with *BRCA1/2* and Lynch mutations, which are the most common germline mutations among adults; over time this space will continue to expand to less common germline mutations and also extend into pediatrics. Table 6 shows clinical trials aimed at precision medicine for pediatric populations with germline cancer mutations.

**Table 5:** Interventional clinical trials for cancer treatment specific for patients with germline cancer mutations in the United States.

**Germline mutation**	**Intervention**	**Cancer**	**Identifier**
*BRCA 1/2*	Talazoparib	Breast cancer	NCT05141708
*BRCA 1/2*	Olaparib	Breast cancer	NCT03150576
*BRCA 1/2*	Olaparib, Palbociclib, Fulvestrant	Breast cancer	NCT03685331
*TP53*	Radiation	SHH-medulloblastoma	NCT02066220
*PTEN*	TAS-117	Solid tumors	NCT04770246
Trisomy 21	Blinatumomab	ALL	NCT03914625, NCT04546399
Trisomy 21	Kymriah	ALL	NCT03876769
Trisomy 21	Chemotherapy	AML	NCT02521493
Multiple germline mutations	Biomarker driven targeted therapy	Pan cancer	NCT02693535

ALL, acute lymphoblastic leukemia; AML, acute myeloid leukemia; SHH, sonic hedgehog.

**Table 6:** Precision medicine matches enrolling pediatric patients with germline cancer mutations in the United States.

**Sponsor**	**Study**	**Identifier**
Dana Farber	The iCat2, GAIN (Genomic Assessment Informs Novel Therapy) Consortium Study	NCT02520713
Massive Bio, Inc.	SYNERGY-AI: Artificial Intelligence Based Precision Oncology Clinical Trial Matching and Registry	NCT03452774
National Cancer Institute	Targeted therapy directed by genetic testing in treating pediatric patients with relapsed or refractory advanced solid tumors, Non-Hodgkin Lymphomas, or histiocytic disorders (the pediatric MATCH screening trial)	NCT03155620
St. Jude	Familial Investigations of childhood cancer predisposition	NCT03050268
Wake Forrest University Health Sciences	PEACH TRIAL- Precision medicine and adoptive cellular therapy (PEACH)	NCT04837547

A fascinating therapeutic intersection between germline and somatic mutational profiling involves repurposing agents known to target somatic mutations for the oncology management of those with germline cancer predisposition. Such a basket trial that is specifically inclusive of those with germline cancer predisposition mutations is the American Society of Clinical Oncology-sponsored study: Testing the Use of Food and Drug Administration (FDA) Approved Drugs That Target a Specific Abnormality in a Tumor Gene in People With Advanced Stage Cancer (TAPUR) (NCT02693535). Similarly, we may not need to rely on as yet undeveloped therapies by moving targeted agents, such as immunotherapies, that are currently reserved for second- or later-line treatment to the upfront settings for those with germline cancer mutations.

Borrowing from our Trisomy 21 model, in addition to identifying novel therapies or repurposing known agents in the treatment of cancers diagnosed among these populations, we may also improve outcomes by studying the effects of standard of care therapies. Aggregate data with short- and long-term follow-up would enable us to truly understand the burden of standard of care chemotherapy and radiotherapy on those with germline cancer predisposition. By understanding which specific agents have increased toxicity profiles for each germline cancer mutation and by developing supportive care interventions to mitigate that toxicity accordingly, such studies would provide insight into rationally modifying therapy with the tools we have already. For example, the recognition that methotrexate imparts greater toxicity (86) to those with Trisomy 21 when used for leukemia treatment led to dose adjustment and increased use of leucovorin (87) to neutralize the drug. Similar reevaluation of our current therapies and supportive care strategies across cohorts of oncology patients who harbor a germline cancer predisposition mutation may offer strategies to decrease morbidity and mortality now. Table 7 shows the current registries and biobanks enrolling pediatric patients with germline cancer mutations.

**Table 7:** Germline cancer registries and biobanks enrolling pediatric patients in the United States.

**Cancer registries and biomarker studies**	**Study**	**Identifier**
*BAP1*	Long term follow-up of mesothelioma patients and their family members with germline mutations in *BAP1* and other genes	NCT03830229
*BAP1*	Frequency and clinical phenotype of *BAP1* hereditary predisposition syndrome	NCT04792463
*BRCA ½*	Triple negative blood cancer and hereditary breast and ovarian cancer mutation carrier registry	NCT02302742
*DICER1*	International ovarian and testicular stromal tumor registry	NCT01970696
*DICER1*	*DICER1*-related pleuropulmonary blastoma cancer predisposition syndrome: a natural history study	NCT01247597
*DICER1*	International PPB/*DICER1* registry	NCT03382158
*NF1*	Neurofibromatosis registry portal	NCT01885767
*TP53*	Li-Fraumeni Syndrome/*TP53* Biobank	NCT04367246
*PTEN*	Natural history study of individuals with autism and germline heterozygous *PTEN* mutations	NCT02461446
*VHL*	Von Hippel-Lindau: Clinical manifestations, diagnosis, management and molecular bases of inherited renal and other urologic malignant disorders	NCT00001238
Adrenocortical tumor	International pediatric adrenocortical tumor registry	NCT00700414
Bone marrow failure	Cancer in inherited bone marrow failure syndromes	NCT00027274
Chordoma	Genetic clues to chordoma etiology: a protocol to identify sporadic chordoma patients for studies of cancer-susceptibility genes	NCT01200680
Gastric cancer	Hereditary gastric cancer syndromes: an integrated genomic and clinicopathologic study of the predisposition to gastric cancer	NCT03030404
Lung cancer	Genetic susceptibility to lung cancer in never smokers	NCT00597636
Malignant peripheral nerve sheath tumors	Multi-institutional registry for malignant peripheral nerve sheath tumors	NCT03141021
Melanoma	A model for genetic susceptibility: melanoma	NCT00591500
MEN syndrome	Registry for multiple endocrine neoplasia syndromes: MEN1/MEN2	NCT03048279
MEN syndrome	Variables that are correlated to developing multiple endocrine neoplasia and pancreatic neuroendocrine tumors	NCT03053999
Neurofibromatosis	Neurofibromatosis registry portal	NCT01885767
Pheochromocytomas and paragangliomas	Genetic analysis of pheochromocytomas, paragangliomas and associated conditions	NCT03160274
Thyroid cancer	Hereditary risk factors for thyroid cancer	NCT02747888
Rhabdomyosarcoma	Genetic mutational analysis of saliva or buccal mucosa samples from patients with embryonal or alveolar rhabdomyosarcoma	NCT03296371
Renal cancer	Hereditary leiomyomatosis renal cell cancer - study of the genetic cause and the predisposition to renal cancer	NCT00050752
Renal cancer	Genetic analysis of Birt Hogg-Dube syndrome and characterization of predisposition to kidney cancer	NCT00033137
Retinoblastoma	Retinoblastoma biomarker study	NCT00342797
Retinoblastoma	RB liquid biopsy biorepository	NCT04959097
Select solid tumors	Genomic structural variation in cancer susceptibility	NCT00996710
Select solid tumors	Germline alterations of tumor susceptibility genes in New York cancer patients	NCT00579514
Rare solid tumors	Natural history and biospecimen acquisition for children and adults with rare solid tumors	NCT03739827
Pan cancer	Childhood cancer predisposition study	NCT04511806
Pan cancer	Clinical, laboratory, and epidemiologic characterization of individuals and families at high risk of cancer	NCT00001163
Pan cancer	Discovering new genetic markers in adults and children who may be at risk for hereditary forms of cancer	NCT03922893
Pan cancer	InAdvance: surveillance, prevention, and interception in a population at risk for cancer	NCT05463796

PPB, pleuropulmonary blastoma; RB, retinoblastoma.

## Oncofertility

Awareness of the presence of a germline cancer predisposition affords the opportunity for family planning, such as egg or sperm banking during early to peak fertile years, ideally prior to the need for cancer treatment, and preimplantation genetic testing (87) to reduce the risk of passing the germline mutation on to the next generation. As the egg yield is highest per cycle of ovarian stimulation during peak fertility, banking eggs during that window minimizes the aggregate hormonal exposure of multiple cycles of ovarian stimulation. The impact of high, cumulative hormonal exposure due to multiple cycles of ovarian stimulation for women at risk for hereditary breast and ovarian cancer is not well-described. Also, premature ovarian failure is more common among women with Lynch Syndrome and *BRCA* mutations (94), further supporting the need for egg harvesting during peak fertile years. Additionally, as germline cancer mutations can be associated with genetic instability during cellular division, reproduction may be especially impacted. Theoretically, there may also be an increased risk of aneuploidy (95, 96), leading to increased risk of recurrent miscarriages and decreased fertility among women with germline cancer mutations. Current guidelines for aneuploidy testing are for women over the age of 35 years, but this may underserve women with germline cancer mutations, who may benefit from aneuploidy testing regardless of age (95).

Much of this area is unchartered, due to the low diagnosis rates of germline cancer mutations and barriers to accessing fertility care. While financial assistance is overall limited, some support may be available for those with a personal diagnosis of cancer to access fertility treatment or medication at a reduced cost (97), and potentially through insurances under state-specific mandated coverage for egg or sperm banking for those with germline cancer mutations. As our awareness of the burden of germline cancer predisposition improves, our ability to support previvors and survivors will concurrently improve.

## Pharmacogenomics in pediatric oncology

Pharmacogenomics is another exploratory space that is growing in parallel with the field of germline cancer genetics. It is established that polymorphisms and/or mutations in genes involved in drug metabolism can impact the pharmacokinetics of various medicines, including chemotherapies, immunosuppressants, and supportive care medications in pediatrics (98). While people who have germline predisposition to therapy intolerance are not more likely to develop a primary malignancy, they are more likely to have adverse events from the management of a cancer diagnoses and may be at increased risk of developing a secondary malignancy, such as increased risk of brain tumors and AML among survivors of childhood ALL who are treated with mercaptopurine and have low-activity *TMPT* genetic polymorphisms (98, 99). Studying the specific toxicities experienced in these populations to various chemoimmunotherapeutic agents similarly has the potential to rationally design interventions that decrease morbidity, mortality, and potentially risk of developing a secondary malignancy. Many germline cancer mutations are involved in maintaining DNA integrity and are not necessarily known to be associated with the metabolism of enzymes, though the impact of coinheritance, downstream effects, or epigenetics is not well-understood. The presumptive etiology for increased methotrexate toxicity observed among oncology patients with Trisomy 21 involves altered metabolic activity of the folate pathway due to enzymes encoded on Chromosome 21 (100). Additionally, inheritance patterns for cancer predisposition and therapy intolerance are not mutually exclusive and an individual patient may coinherit predispositions to both cancer and therapy intolerance concurrently. This may compound the toxicity profile leading to a more fulminant course or higher risk of late effects. Therefore, while consideration should be given to assessing pharmacogenomic polymorphisms among all cancer patients, there should be an especially low threshold for testing those with cancer predisposition.

## Limitations to cancer testing

### Operational considerations

Pediatric oncologists infrequently test for potential germline cancer mutations (63), even when there is a family history of cancer or somatic cancer findings suggestive of a germline mutation. Factors underlying lack of testing are multifactorial and include: lack of knowledge in a continuously evolving field, difficulty in obtaining financial coverage, lack of resources in an overburdened healthcare system, and perception of adding more distress to an already burdened family (63).

Cancer predisposition testing of those who carry a cancer diagnosis requires the clinical expertise to order the correct test and interpret the results, as well as affiliation with a laboratory that has expertise in processing the requisite samples. Certain scenarios may require skin fibroblast testing (101), such as germline testing a patient with active leukemia, while others are sufficiently addressed with blood or saliva specimens. Appropriate testing may require sequencing, imprinting analysis, or loss of heterozygosity studies; or it may require multiple anatomic site testing in instances of potential mosaicism or reversion mutations. Additionally, genetic cancer mutation panels vary and the ordering physician must carefully consider the best choice or choices of tests to appropriately capture the clinical scenario. Furthermore, some companies that specialize in cancer mutation testing may not report a germline result as the vast majority of current precision medicine-based clinical trials focus on somatic testing; these tests often require a matched “normal” sample, such as a buccal swab or skin fibroblast culture, used to cancel the germline results from the somatic testing report. For those sequencing platforms, a potential germline cancer mutation may therefore be removed from the reports.

Furthermore, reimbursement for these tests is not standardized within and across insurance companies. Variability in reimbursement within a given company may limit testing options or require the ordering physician to hold specific subspecialty credentials, while other companies may not cover critical panels or specific tests in pediatric populations at all. While the National Cancer Center Network (NCCN) guidelines carry significant weight for insurance reimbursement, they are more focused on adult populations. Standardized insurance coverage for germline cancer predisposition testing would improve testing access, as it would allow financial gatekeepers to be more certain of anticipated reimbursement.

The downstream impact of ambiguous reimbursement leads cancer predisposition testing to be deprioritized on health care resource utilization agendas. To best identify who to test, a thorough family history, including a pedigree analysis, is required, (102) yet this would necessitate training on how to obtain such a pedigree and valuable clinic time to conduct the analysis, or corresponding attention by and reimbursement for a cancer genetic counselor or geneticist. To best care for children and young adults diagnosed with a germline cancer mutation, multidisciplinary teams that are inclusive of pediatric oncology subspecialists, cancer geneticists or genetic counselors, social workers, case managers, and psychologists or psychiatrists with dedicated expertise in cancer predisposition are necessary. Variable insurance reimbursement coupled with lower absolute number of pediatric patients relative to adult patients with germline cancer mutations lead to decreased expertise and support for the necessary infrastructure to identify and serve this population.

The siloization that occurs in the wake of reduced access to multidisciplinary care may lead children and adolescents with genetic cancer predisposition to be underserved. Depending on local practices, the medical home for a previvor may exist with a general pediatrician or a geneticist, neither of whom carry the expertise to manage an oncologic diagnosis, or a general oncologist, who may not have the depth of experience to diagnose and manage the full spectrum of potential cancers a previvor may develop. If the patient develops a cancer and their medical home moves to a subspecialized pediatric oncologist, that physician may not have the expertise to effectively tailor therapy based on the given germline mutation or to effectively navigate subsequent oncologic diagnoses that are outside their scope of expertise. A potential intervention to improve germline cancer predisposition literacy among practicing pediatricians, geneticists, and oncologists is through accrediting board-mandated Continuing Medical Education (CME) modules.

At the intersection of testing limitations that lead to low diagnosis rates and lack of expertise in this subject field lies a marginalized population in an overwhelmed health care system. The strain on these supports is further exacerbated by the current SARS-CoV-2 2019 pandemic. Reports from the start of the pandemic anticipated an increased rate of cancer-associated death due to resource limitations leading to later stage oncology diagnoses (103). Post-pandemic, resources are expected to remain limited as large swaths of the population and health care industries may be affected by long COVID, endemic COVID, opportunistic infections, and/or other late effects. With all these barriers to testing and post-diagnosis care delivery, this population remains marginalized.

### Ethical considerations

Autonomy and consent remain critical topics in pediatric germline cancer predisposition testing (104). Acquiring information about the potential for future illnesses has implications for a child's ability to develop their relationship with their own body, challenges young coping mechanisms, may not lead to healthy decision-making and can put them at risk of facing discrimination. While the Genetic Information Nondiscrimination Act (GINA) of 2008 is intended to safeguard an individual with a germline cancer predisposition from health insurance and employment discrimination, laws may be vulnerable due to partisan views on funding priorities and employee protections. Additionally, there are no protections against social discrimination, which may impact opportunities for career growth, leading to disadvantaged financial states, and opportunities for social support, leading to isolation with collateral effects on mental health (105).

There are ongoing efforts to optimize return of cancer predisposition testing results to pediatric cancer patients whose germline cancer mutation or VUS was suggested incidentally by somatic testing or identified on exploratory germline analyses. For example, St. Jude is conducting a study to analyze the psychosocial impact of return of germline results on the patient and family among children who already carry a cancer diagnosis (NCT04848142). Inherent across these studies and interactions, is respect for the patient's autonomy as perceptions of and decisions to receive a germline cancer predisposition diagnosis vary significantly across a cohort of people (106). Current recommendations are to inform the patient and/or guardian that germline results may be identified and ask if and how they want the information shared with them (107, 108). Should they decide to be informed of the test result, key components of these conversations are not simply return of the result, but the mechanism of delivery and the preparation of the family to receive and understand the findings (108). The knowledge and comfort of the provider in discussing the test itself, potential pitfalls, and interpretation are foundational to conveying critical information.

These considerations also extend to the management of children who have not been diagnosed with cancer but have a high risk of germline cancer predisposition, such as those with a confirmed pathogenic mutation in a first-degree relative. In these instances, the decision-making revolves around the risk-benefit ratio for germline cancer predisposition testing and takes into consideration the median age of first cancer onset associated with the given mutation. When the cancer onset tends to occur during the pediatric age range, then testing is considered worthwhile. This paradigm, however, does not consider if preventative opportunities implemented during the pediatric age range could decrease the likelihood of cancer onset in adulthood. Crucially, awareness of an increased cancer risk encourages a previvor to learn what is normal for their body and for all involved, physician and previvor/family alike, to diligently follow up any abnormal finding or persistent symptom; this can overall promote earlier detection of cancer and may improve survival outcomes.

Additionally, awareness of a germline cancer mutation may provide impetus to follow recognized healthy lifestyles, such as maintaining a healthy weight, avoiding tobacco and heavy alcohol use, relocating to areas of decreased radon or nuclear/industrial waste exposure, and increasing uptake of anti-cancer vaccines such as the HPV vaccine. Beyond known cancer risk-reducing interventions that are broadly applicable, further studies can determine the true benefit of other potential interventions that are more targeted, such as early non-steroidal anti-inflammatory drug (NSAID) use to mitigate risk of developing colon cancer among those with hereditary gastrointestinal cancer predisposition (109, 110) or early oral contraceptive use to mitigate the risk of developing endometrial cancer among those with hereditary breast and ovarian cancer predisposition or Lynch syndrome (111). Table 8 shows available clinical trials focused on cancer prevention among adult previvors. Additionally, awareness of a germline cancer mutation can help with professional planning, encouraging those with such a mutation to select jobs or career paths with more insurance stability or consistently maintaining self-funded health insurance. Furthermore, as discussed in the oncofertility section, awareness of a germline cancer predisposition allows for family planning opportunities, which, given that reproductive potential begins in adolescence, brings the age of testing to adolescence as well. Of note, it is important to recognize that the benefit of these options may be limited by a previvor's access to resources, which may be hampered by the compounded effects of depleted generational wealth from the impacts of cancer morbidity and mortality in the family tree and depleted personal wealth from the impacts of social marginalization. Therefore, these recommendations for lifestyle changes, exposure mitigation strategies, career planning to ensure insurance stability, and family planning may not be accessible to many individuals, and other support may be needed to manage cancer risk among vulnerable previvors.

**Table 8:** Interventional clinical trials for cancer prevention among patients with germline cancer mutations in the United States.

**Germline mutation**	**Intervention**	**Cancer**	**Identifier**
*BRCA*	INO 5401 vaccination	Breast	NCT04367675
*BRCA1*	Denosumab	Breast	NCT04711109
*BRCA1/2, BRIP1, RAD51C, RAD51D*	Delayed oophorectomy	Ovarian	NCT05287451
*BARD1, BRCA1/2, BRIP1, EPCAM, MLH1, MSH2, MSH6, PALB2, PMS2, RAD51C, RAD51D*	Delayed oophorectomy	Ovarian	NCT02760849
*ATM, BRCA1/2, CDKN2A, EPCAM, MLH1, MSH2, MSH6, PALB2, PMS2*,	Mutant KRAS -targeted long peptide vaccine	Pancreatic	NCT05013216
*MLH1, MSH2/EPCAM, MSH6, PMS2*	Nous-209 vaccine	Colon	NCT05078866
*MLH1, MSH2/EPCAM, MSH6, PMS2*	Omega 3 fatty acids	Colon	NCT03831698
*MLH1, MSH2/EPCAM, MSH6, PMS2*	Combination of vaccines	Pan cancer	NCT05419011

The intergenerational impact of a germline cancer predisposition mutations may also be felt even in the absence of a confirmed diagnosis. Current germline testing criteria have high points of entry and are based on a “sacrificial lamb” model, whereby sufficient numbers of first- and/or second-degree relatives must be affected by cancer to trigger evidence-based germline testing and insurance coverage (term “sacrificial lamb” used in this context was coined by Heather Hampel, MS, CGC in a personal communication). Furthermore, family history-based testing criteria presumes access to sufficient historical data, yet there is a high likelihood of limited family history due to small families, unknown family history, and/or death in the family from a competing non-oncologic cause (112). Additionally, in the context of cancer-associated death of affected family members, the ability to access histological specimens or key pathology information that can inform germline genetic testing is restricted, which can further create barriers to appropriate cancer predisposition evaluation. Finally, family-history based testing strategies do not translate well into pediatrics as the majority of the proband's first- and second-degree relatives may be young, leading to decreased ability for family history to capture mutations associated with adult-onset cancers (50). There are also complex ethical issues associated with predictive genetic testing for adult-onset cancers in a pediatric population.

Even with a well-documented family history, germline cancer predisposition testing guidelines may be limiting. Various guidelines include those put forth by the American Society of Breast Surgeons (ASBS) for hereditary breast and ovarian cancer (113), revised Bethesda criteria for Lynch syndrome (114), and, more recently, the National Cancer Center Network (NCCN) for hereditary breast, ovarian, prostate and pancreatic cancer (115); and hereditary colon cancer (116). Individuals who meet criteria for testing through ASBS or the revised Bethesda criteria, may not be captured by the NCCN, leading to gaps in access. For example, Beitsch et al. identified ~50% of 1,000 breast cancer patients who were confirmed to have a pathogenic or likely pathogenic variant, but would not have met NCCN 2017 criteria for germline testing (117). Appropriately, the guidelines are not static and expert panels across these bodies regularly review new data and revise recommendations, with the most updated NCCN guidelines published in 2022 (115, 116).

Liberalizing germline cancer predisposition testing criteria would enhance access to potentially life-changing results for both the patient and their family. The detection of a germline cancer mutation allows for cascade family testing and, through prophylactic procedures and interventions of newly diagnosed family, can save the lives of a proband's first and/or second-degree relatives and allow them to navigate life with their family present as opposed to their family members dying early of cancer (118). As testing becomes more accessible and more are diagnosed with a germline cancer predisposition, the stigma associated with the diagnosis can abate leading to reduced discrimination and improved access to support. Destigmatization also allows improved access to information as affected family members may be more willing to share their germline and/or cancer diagnoses.

While strict germline testing criteria do help identify groups of people with a higher likelihood of having a positive result, there are systemic gaps that are more pronounced for pediatric patients, and gatekeeping the diagnostic test both enforces a sacrificial lamb approach that carries a heavy emotional burden on the remaining family and perpetuates marginalization of this population. On the opposite end of the spectrum, primary screening of the pediatric population, in the form of mass testing, is beyond the scope of this article, but has intriguingly been discussed in the context of newborn screening (119, 120). Across all germline cancer predisposition testing strategies, the ethical principles of autonomy, justice, beneficence, and nonmaleficence must be considered (121).

## Future directions

There are many challenges and opportunities remaining to realize the potential of precision medicine among pediatric populations with germline cancer predisposition mutations. As the motivating example of therapy modification among children with Trisomy 21 diagnosed with AML has shown, dramatic improvements in cancer care and outcomes are possible. To reach this potential for all pediatric patients, more research is needed to (1) estimate the prevalence of germline predisposition among childhood cancer patients; (2) expand known associations between clinical and histological features and underlying germline cancer predisposition mutations; (3) determine the settings in which risk-adaptive therapy is appropriate, considering the high risk of second malignancies among patients with germline predisposition; and (4) expand the diversity of enrollment of children into therapeutic and research protocols to enable broader generalizability of research findings.

## Author contributions

SS and EM: article concept and design, critical revisions of the manuscript for important intellectual content. SS: drafting of the manuscript. All authors listed have made a substantial, direct, and intellectual contribution to the work and approved it for publication.

## Funding

This work was supported by the Children's Cancer Research Fund (Minneapolis, MN).

## Conflict of interest

The authors declare that the research was conducted in the absence of any commercial or financial relationships that could be construed as a potential conflict of interest.

## Publisher's note

All claims expressed in this article are solely those of the authors and do not necessarily represent those of their affiliated organizations, or those of the publisher, the editors and the reviewers. Any product that may be evaluated in this article, or claim that may be made by its manufacturer, is not guaranteed or endorsed by the publisher.
